# Is reduced dentition with and without dental prosthesis associate with oral health-related quality of life? A cross-sectional study

**DOI:** 10.1186/s12955-019-1149-2

**Published:** 2019-05-03

**Authors:** Raquel Conceição Ferreira, Ichiro Kawachi, João Gabriel Silva Souza, Fernanda Lamounier Campos, Loliza Luiz Figueiredo Houri Chalub, José Leopoldo Ferreira Antunes

**Affiliations:** 1 School of Dentistry, Federal University of Minas Gerais, 6627 Antonio Carlos Avenue, Belo Horizonte, MG 31270-901 Brazil; 2 Harvard T.H. Chan School of Public Health, Harvard University, 677 Huntington Avenue, Boston, MA 02115 USA; 3 Piracicaba Dental School, University of Campinas, 901 Limeira Avenue, Piracicaba, SP 13414-903 Brazil; 4 School of Public Health, University of São Paulo, 715, Dr. Arnaldo Avenue, São Paulo, SP 01246-904 Brazil

**Keywords:** Tooth loss, Quality of life, Oral health, Dental health surveys, Epidemiology, Dental prosthesis

## Abstract

**Background:**

Oral health-related quality of life (OHRQoL) has important implications for the clinical practice of dentistry and dental research and should contribute to professional judgment about restorative treatments and prosthetic replacement in patients who had reduced dentitions. The aim was to compare the OHRQoL among adults (35–44 years) categorized according to different definitions of reduced dentition and considering the use (or non-use) of dental prosthesis.

**Methods:**

This study used data from a probabilistic sample of adults in Sao Paulo, Brazil, 2015. OHRQoL was based on none items of *Oral Impacts on Daily Performance* (OIDP) index, as prevalence (at least one impact) and extent (the number of items with non-zero score). We used different criteria to assess dentition status: (1) Shortened Dental Arch (SDA): having 3–5 natural occlusal units (OUs) in posterior teeth and intact anterior region; (2) hierarchical functional classification system: a five-level stepwise classification of dentition; and (3) presence of ≥21 teeth. The use or nonuse of dental prosthesis was recorded. Negative binomial regression models involved the adjustment for social determinants of health.

**Results:**

Nearly half (53.1%) of the 5753 participating adults had at least one oral health issue impacting OHRQoL. OIDP prevalence in adults with SDA did not differ from those with more OUs (PR = 1.02; 95%CI 0.91–1.13). Individuals with non-functional dentition had worse OHRQoL regardless of their use of a dental prosthesis. Adults with fewer than 21 remaining teeth, ranked significantly higher in OIDP extent, regardless of dental prosthesis use (PR = 1.38; 95%CI 1.16–1.63 with prosthesis; PR = 1.62; 95%CI 1.19–2.20 without dental prosthesis).

**Conclusions:**

Individuals with more missing teeth reported worse OHRQoL regardless of using a dental prosthesis. Preserving a functional dentition, even with missing teeth, is compatible with OHRQoL.

## Background

The FDI World Dental Federation launched a new definition of “oral health” in 2016, as part of the organization’s strategic plan (Vision 2020): “Oral health is a fundamental component of health”, and “is influenced by the person’s changing experiences, perceptions, expectations, and ability to adapt to circumstances” [1]. Furthermore, oral health “is multifaceted and includes the ability to speak, smile, smell, taste, touch, chew, swallow, and convey a range of emotions through facial expressions with confidence and without pain, discomfort, and disease of the craniofacial complex” [1].

The concept of the oral health-related quality of life (OHRQoL) is in line with this new definition because it is a multidimensional construct representing subjective assessments of how much oral conditions affect a person’s daily life [2]. OHRQoL has important implications for the clinical practice of dentistry and dental research. The impact of dental disease and its treatment on quality of life has been increasingly considered when assessing health status [3].

Tooth loss is a critical indicator of oral health status [4, 5] and OHRQoL [6]. Several studies reported the association between reduced dentition and OHRQoL [7–10], as well as the performance of oral functions [11, 12]. Retaining a reduced dentition can be compatible with OHRQoL when dental status preserves some characteristics that are required for performing oral functions and esthetics (i.e., presence of anterior teeth and occlusal pairs) [13]. The loss of posterior teeth seems to have a lower negative impact on OHRQoL than the loss of anterior teeth when some occluding pairs remain [14].

Different criteria to define reduced dentition have been proposed. A Shortened Dental Arch (SDA) is defined as keeping intact anterior teeth plus a functional number of occlusal contacts among posterior teeth [12]. The World Health Organization (WHO) stated that keeping a functional, esthetic, and natural dentition of 21 or more teeth during lifetime, as well as not needing a dental prosthesis, should be treatment goals for oral health [15]. This WHO definition takes into account only the number of natural remaining teeth. This cutoff of 21 teeth was also used by Hobdell et al. [15] to define global goals for oral health. To add other criteria, such as occlusion, Nguyen et al. [16] proposed a hierarchical dental functional classification system to assess dentition functionality based on the number and type of natural teeth and the number of occlusion pairs. These definitions are based on the number of teeth, their location, and function. Therefore, when differently defined, the status of the dentition may have different impacts on oral health quality of life.

Epidemiological studies have evaluated the association between reduced dentition and patient-centered outcomes, such as OHRQoL [9, 10, 17], general quality of life [18], and satisfaction with dentition [9, 19]. People with SDA reported no more negative impacts on OHRQoL [10, 17] and general quality of life [18] compared to individuals with more posterior occluding pairs. Individuals who met all criteria defined by the hierarchical dental functional classification system have greater satisfaction with their mouths [19] as well as higher OHRQoL [7].

The growing interest in assessing the functionality of a reduced dentition, as defined by different criteria, suggests that not all missing teeth demand a prosthetic replacement. At the population level, treatment plans involving removable or fixed dental prosthesis for people with shortened dentitions should move towards preventive and restorative procedures aimed at maintaining functionality with their remaining natural dentition [10]. Few epidemiological studies investigated the association between reduced dentition and the extent of oral impacts [10, 17]. Also, few studies concurrently assessed the association between reduced dentition and OHRQoL, considering the use of dental prosthesis [7, 19]. The effect of prosthetic rehabilitation on OHRQoL can vary when the remaining natural teeth are more or less favorably distributed to perform the oral functions and esthetics. Also, the assessment of reduced dentition by different criteria of dental status may contribute to evaluating whether the maintenance of natural teeth is compatible with oral health-related quality of life, even in the absence of dental prostheses. Accordingly, this study aimed to compare the OHRQoL among adults (35–44 years) categorized according to different definitions of reduced dentition and considering the use (or non-use) of dental prosthesis.

## Methods

### Ethical aspects

The study was approved by the local Research and Ethics Committee from the University of Campinas (CAEE no. 46788215.9.0000.5418). All participants provided written informed consent.

### Survey sampling design and sample

The cross-sectional data in this study was part of the *São Paulo Oral Health Survey – 2015* [20], which followed methodologic criteria established by the World Health Organization (1997) [21]. São Paulo (SP) is the most populous state in Brazil, with 44.5 million inhabitants, or 21.7% of the Brazilian population. Participants were selected following a multistage cluster sampling design with probability proportional to population size. Individuals aged 15–19, 35–44 and 65–74 years old were interviewed and examined in their homes. Further details of the sampling design were previously reported [20]. Examinations and interviews were conducted by previously trained dentists and recording clerks. For each examiner, the minimum acceptable kappa statistic was 0.65 for all conditions observed (dental decay, periodontal disease, use and need of dental prosthesis) during the dental examination. This study specifically focused on adults (35–44 years old). Edentate adults who used bimaxillary complete dentures were excluded because our interest was to evaluate the effect of the dental prosthesis combined with the presence of remaining teeth, as classified by different definitions of dentition status.

### Study outcome and covariates

Our outcomes were defined based on the *Oral Impacts on Daily Performance* (OIDP), an instrument to evaluate oral health-related quality of life [22]. We used nine questions [20], addressing problems caused by teeth during the previous six months, on the following aspects of daily life: (1) eating food, (2) cleaning teeth, (3) becoming easily upset (emotional state), (4) enjoy social contact, (5) doing light physical activities, (6) speaking clearly, (7) smiling, laughing and showing teeth without embarrassment, (8) carrying out work, and (9) sleeping/relaxing. The option of answers to each one of the nine questions was “no” (score 0) or “yes” (score 1). We have defined two outcomes: i) OIDP prevalence: the proportion of individuals who answered affirmatively to at least one of the nine questions. ii) OIDP dichotomous frequency score (OIDP extent): the sum of items with a non-zero score in the nine items (range: 0–9). A previous Brazilian study supported the unidimensionality and the use of an overall score based on dichotomous items of OIDP [23].

Our main exposure variable of interest was dentition status, classified according to three distinct criteria based on the number and location of present teeth. The dental condition was evaluated using the Decayed Missing Filling Teeth Index (DMFT). We also assessed the use of dental prosthesis of any type [fixed (FDP) or removable partial dental prosthesis (RDP)].

(1) *Shortened dental arch* (SDA), defined as having an intact anterior region and 3–5 natural Occlusal Units (OUs) [12]. In this definition, an occlusal pair of premolar teeth counts one OU, whereas an occlusal pair of molar teeth counts two OUs [24]. Participants with intact anterior region were classified into the following categories: (i) > 5 OUs, no dental prosthesis, (ii) > 5 OUs, with dental prosthesis, (iii) 3–5 OUs, no dental prosthesis (corresponding to SDA), (iv) < 3 OUs, no dental prosthesis, (v) ≤ 5 OUs, with dental prosthesis [10, 17].

(2) *Hierarchical dental functional classification system,* defined by a dichotomized five-level stepwise branching hierarchy based on conditions (esthetics and occlusion) that reflect functionality: sequential presence of one tooth in each arch (dentition level), ≥ 10 teeth in each arch (arch level), 12 anterior teeth (anterior level), 3 or 4 posterior occluding pairs (POPs) of premolars (premolar level), and (5) ≥ 1 POPs of molar bilaterally (molar level) [16]. Individuals who met all these conditions were classified as having functional dentition. Subsequently, the following categories were used: (i) functional dentition, no dental prosthesis; (ii) functional dentition, with dental prosthesis; (iii) no functional dentition, with dental prosthesis; (iv) no functional dentition, no dental prosthesis. A complete description and evaluation of this system in Brazil were previously reported [25].

(3) *WHO Criteri*a *for functional dentition*: Functional dentition was defined by the presence of 21 or more teeth, a criterion originally proposed by the WHO [15]. The following categories were considered: (i) > 21 teeth, no dental prosthesis; (ii) > 21 teeth, with dental prosthesis; (iii) < 21 teeth, with dental prosthesis; and (iv) < 21 teeth, no dental prosthesis.

The association between OHRQoL and dentition status was adjusted for a range of covariates [26, 27], including socio-demographic characteristics (sex, race/skin color, age group (35–39; 40–44 years), household income and education. Although the age range is narrow, we controlled for age-group as there may be subtle differences by age in the association between tooth loss and OHRQoL and previous studies showed differences in prevalence of functional dentition between the ages range 35–39 and 40–44 years old [28, 29]. Skin color refers to the classification adopted in demographic censuses performed in Brazil: whites, blacks, browns, yellows, and Amerindians. Household income (up to R$ 500.00; from R$ 501.00 to R$ 2500.00; and over R$ 2501.00) used Reals, the official currency in Brazil (one US dollar was 3,80 Reals during the period of data gathering). Education (number of years of formal education) was classified as less than four years (insufficient education), 4–7 (incomplete fundamental education), 8–10 (complete fundamental, incomplete secondary education), and 11 or more (complete secondary, incomplete university education, university).

We also assessed and adjusted the analysis for the following covariates: the use of dental services, dental conditions, and social capital, self-reports of perceived need for dental treatment and dental prosthesis. As refers to dental services, we assessed the time since the last dental visit (< 12 months, between 1 and 2 years, > 2 years, did not visit). Dental conditions refer to dental caries (DMFT index), periodontal conditions (bleeding on probing, dental calculus, shallow (4–5 mm) and deep (> 6 mm) pockets), and dental pain (previous six months). Social capital was evaluated according to Grootaert et al., (2004) [30].

### Statistical analysis

A descriptive analysis was conducted to summarize the distribution of the sample according to the covariates of the study. Negative binomial regression was selected to assess the association between dentition status and the outcomes on OHRQoL, due to the overdispersion of OIDP extent. For OIDP prevalence (event not rare), we used the same model, which corresponds to Poisson regression in the absence of overdispersion, and because we aimed at estimating prevalence ratios. The multiple final models included covariates with *p*-value < 0.05. The statistical analysis used Stata 15.1 (Stata Corp., College Station TX, USA, 2018) taking into account the complex survey design and sample weights.

## Results

Overall, 6051 adults participated in the survey. Seventy-six individuals (1.37%) were excluded because they used bimaxillary complete dentures. No significant differences of OIDP prevalence (*p* = 0.07) and OIDP extent (0.258) was observed for those who use or do not use bimaxillary complete dentures. A total of 5753 did not have missing data to OIDP, use of dental prosthesis and dental status variables and were included in the analysis, thus accounting a non-response rate of 4.92%. Sample characteristics according to evaluated variables are shown in Table 1.

**Table 1:** Characteristics of the adults. State of Sao Paulo, Brazil, 2015

Covariates	Total sample	
	n	Frequency^a^
Personal characteristics and socioeconomic conditions		
Sex		
Male	1862	30.7 (27.5, 33.1)
Female	3891	69.3 (65.9, 72.5)
Race/Skin color		
White	3571	60.4 (56.1, 64.5)
Brown	1672	30.3 (26.9,33.8)
Black	445	8.4 (6.9, 10.1)
Others	65	0.9 (0.6, 1.5)
Age group		
35,39	2970	51.8 (49.7, 53.9)
40,44	2783	48.2 (46.1, 50.3)
Income		
> USD$ 658,00	1273	22.6 (19.4, 26.2)
USD$ 132,00 to 657,00	3635	70.5 (67.1, 73.8)
< USD$ 131,00	274	6.85 (5.41, 8.65)
Education (Years of study) ^b^		
> 11	2630	46.3 (42.3, 50.4)
8–10	1153	21.5 (19.1, 24.2)
4–7	1368	25.4 (21.9, 29.2)
< 4 years	395	6.8 (5.55, 8.25)
Health services		
Time since last dentist visit		
< 12 months	3146	52.3 (48.7, 55.8)
1–2 years	1451	26.5 (24.3, 28.9)
> 2 years	993	20.6 (17.4, 24.3)
Has not visited	52	0.5 (0.3, 0.9)
Dental conditions		
DMFT		
0–10	1261	24.1 (21.3, 27.1)
11–20	3122	52.9 (50.5, 55.3)
> 21	1370	23.1 (20.9, 25.4)
Untreated caries		
0	2554	41.6 (38.1, 45.3)
1–3	2035	36.5 (34.6, 38.4)
4–6	684	13.5 (11.3, 16.1)
> 7	103	8.4 (6.8, 10.3)
Bleeding on probing^b^		
No	3222	55.8 (51.7, 59.8)
Yes	2419	44.2 (40.2, 48.3)
Dental calculus^b^		
No	2480	42.7 (38.9, 46.5)
Yes	3161	57.3 (53.5, 61.0)
Presence of periodontal pockets^b^		
No	4175	73.2 (68.7, 77.2)
Shallow pockets	1212	22.4 (18.8, 26.6)
Deep pockets	254	4.4 (3.4, 5.6)
Symptoms status		
Dental pain^b^		
No	3897	67.6 (64.6, 70.4)
Yes	1808	32.4 (29.6, 35.4)
Capital Social		
Probability of cooperation^b^		
Much or relatively likely	4122	69.8 (66.2, 73.3)
Neither likely nor unlikely	598	13.0 (9.2, 18.0)
Much or relatively unlikely	1031	17.2 (14.5, 20.2)
Feeling of safe		
Much or relatively safe	3368	57.1 (51.5, 62.6)
Neither safe nor unsafe	684	12.1 (10.1, 14.3)
Much or relatively unsafe	1699	30.8 (25.4, 37.2)
Self-perception of happiness		
Much or relatively happy	5026	86.9 (84.6, 89.0)
Neither help nor unhappy	457	7.9 (6.4, 9.6)
Much or relatively unhappy	263	5.2 (4.3, 6.3)
Oral health perception		
Self-perception of need of dental treatment		
No	1169	81.77 (79.4, 83.9)
Yes	4534	18.23 (16.1, 20.6)
Self-perception of need of dental prosthesis		
No	4796	16.3 (13.1, 20.0)
Yes	755	83.7 (79.9, 86.9)

^a^Proportion (and 95% confidence intervals) of individuals regarding studied variables; estimates considered weighting and complex sampling design. ^b^The following variables have missing values: Education: 207 missing values; Bleeding on probing, Presence of calculus and Presence of periodontal pocket: 112 missing values; Dental pain: 48 missing values; Probability of cooperation: 2 missing values; Feeling of safe: 2 missing values; Self-perception of happiness: 7 missing values; Self-perception of need of dental treatment: 50 missing values; Self-perception of need of dental prosthesis: 202 missing values

Among those who participated, 53.1% (95% CI: 49.5; 56.6) had at least one oral impact on daily performances. The most affected domains were eating food; smiling, laughing and showing teeth without embarrassment and cleaning teeth or dentures (Table 2). The mean score of OIDP extent was 1.81 (Range: 0–9; 95% CI: 1.64; 1.98). Cronbach’s Alpha of the scale was 0.856.

**Table 2:** Prevalence of oral impacts on daily performance among adults. State of São Paulo, Brazil, 2015 (*N* = 5753)

OIDP subscale scores	n	Affected 95% CI
Eating	3723	37.7 (34.5, 40.9)
Smiling, laughing and showing teeth without embarrassment	1461	26.7 (22.9, 30.9)
Cleaning teeth	1355	26.6 (24.2, 29.1)
Emotional status (becoming easily upset)	1414	25.3 (23.0, 27.6)
Sleeping/relaxing	1201	22.2 (19.8, 24.9)
Enjoy social contact (going out)	769	15.1 (12.9, 17.7)
Speaking clearly	734	14.0 (11.0, 17.7)
Carrying out work	459	8.9 (7.3, 10.7)
Doing light physical activity	328	5.6 (4.4, 7.2)

95% confidence intervals (CI) in brackets. Estimates considered weighting and complex sampling design

The prevalence of SDA was 7.8%; 46.7% met all criteria of the hierarchical system but did not use dental prosthesis; while 75.0% had > 21 teeth, no dental prosthesis. For the assessment of SDA, adults with ≤5 OUs plus use of dental prosthesis had the highest prevalence (73.5%) of reporting problems in at least one of the nine domains of OIDP. Interestingly, the same group did not report the highest extent regarding the average score of OIDP problems. The group with the highest extent score (2.9) was adults with < 3 OUs using no dental prosthesis. When we turn to hierarchical dental functional classification, the group with the highest prevalence and extent of OIDP problems was adults lacking functional dentition/using a dental prosthesis (61.9%; mean: 2.6). Lastly, when we look at WHO criteria, OIDP prevalence and extent was highest in individuals with < 21 teeth and no use of dental prosthesis (68.8%; mean: 3.1) (Table 3).

**Table 3:** OIDP prevalence and extent in adults, according to dentition status. State of São Paulo, Brazil, 2015 (*N* = 5753)

Definitions of dentition status	Total sample		OIDP prevalence		OIDP extent
	n	% (95% CI)	n	% (95% CI)	Mean (95% CI)
Shortened Dental Arch					
> 5 OUs, no dental prosthesis	3471	58.7 (55.9, 61.4)	1503	47.1 (43.3, 50.9)	1.3 (1.2, 1.5)
> 5 OUs, with dental prosthesis	137	2.1 (1.5, 2.9)	64	45.1 (32.3, 58.5)	1.5 (0.9, 2.1)
3,5 OUs, no dental prosthesis (SDA)	487	7.8 (6.8, 9.0)	285	56.5 (49.9, 63.8)	2.1 (1.8, 2.5)
< 3 OUs, no dental prosthesis	197	3.5 (2.9, 4.2)	130	72.7 (64.0, 81.3)	2.9 (2.2, 3.6)
≤ 5 OUs, with dental prosthesis	117	2.1 (1.5, 2.9)	73	73.5 (62.1, 82.4)	2.7 (1.8, 3.6)
No intact anterior region	1344	25.7 (23.8, 28.40)	864	62.1 (57.0, 66.9)	2.6 (2.3, 2.9)
Hierarchical Dental Functional Classification0					
Functional dentition, no dental prosthesis	2805	046.7 (43.8, 49.6)	1163	44.9 (40.6, 49.4)	1.3 (1.1, 1.4)
Functional dentition, with dental prosthesis	95	01.5 (1.0, 2.3)	40	40.0 (25.9, 55.8)	1.3 (0.7, 2.0)
No functional dentition, no dental prosthesis	1887	330.1 (31.1, 35.2)	1091	60.3 (56.6, 63.9)	2.2 (1.9, 2.4)
No functional dentition, with dental prosthesis	966	18.07 (16.9, 20.6)	625	61.8 (54.6, 68.5)	2.6 (2.2, 3.0)
WHO criteria for functional dentition					
> 21 teeth, no dental prosthesis	4452	75.0 (72.3, 77.5)	2094	50.2 (46.7, 53.6)	1.6 (1.4, 1.7)
> 21 teeth, with dental prosthesis	5301	9.6 (8.3, 11.1)	326	63.9 (55.3, 71.8)	2.5 (2.0, 3.0)
< 21 teeth, no dental prosthesis	240	4.8 (3.6, 6.3)	160	68.8 (59.6, 76.8)	3.1 (2.6, 3.6)
< 21 teeth, with dental prosthesis	530	10.6 (9.1, 12.4)	339	56.7 (46.2, 66.5)	2.5 (1.8, 3.2)

Prevalence and extent rate (95% confidence intervals (CI) in brackets). Estimates considered weighting and complex sampling design. *OUs* Occlusal Units, *SDA* Shortened Dental Arch

Table 4 shows the unadjusted association between OIDP (prevalence and extent) and covariates. Age was the only covariate that was not statistically significantly associated with either of our outcomes (Table 4).

**Table 4:** Factors associating with OIDP prevalence and extent in adults. State of São Paulo, Brazil, 2015

Covariates	OIDP prevalence^a^	Unadjusted Prevalence Ratio^b^	OIDP extent^c^	Unadjusted count ratio^d^
Personal characteristics and socioeconomic conditions				
Sex				
Male	44.9 (39.8,50.2)	1	1.4 (1.2,1.7)	1
Female	56.7 (53.0,60.3)	1.26***(1.13,1.41)	2.0 (1.8,2.2)	1.40^***^(1.23,1.60)
Race/Skin color				
White	47.6 (43.6,51.6)	1	1.6 (1.4,1.8)	1
Brown	61.6 (57.2,65.8)	1.29***(1.19,1.40)	2.1 (1.8,2.3)	1.28^***^(1.13,1.45)
Black	61.1 (52.8,68.8)	1.28**(1.11,1.48)	2.3 (1.8,2.7)	1.39^**^(1.14,1.71)
Others	60.5 (44.1,74.8)	1.27 (0.97,1.67)	2.2 (1.5,3.0)	1.37 (0.96,1.94)
Age group				
35,39	52.2 (48.7,55.7)	1	1.8 (1.6,1.9)	1
40,44	54.0 (49.8,58.2)	1.03 (0.97,1.10)	1.9 (1.6,2.1)	1.05 (0.94,1.17)
Income				
> USD$ 658,00	42.4 (37.6,47.5)	1	1.1 (0.9,1.3)	1
USD$ 132,00 to 657,00	55.2 (51.1,59.2)	1.30*** (1.16,1.46)	1.9 (1.8,2.1)	1.70^***^(1.40,2.07)
< USD$ 131,00	64.1 (51.9,74.7)	1.51***(1.22,1.87)	2.7 (1.9,3.4)	2.38^***^(1.68,3.37)
Education (Years of study)				
> 11	46.3 (41.7,50.9)	1	1.4 (1.2,1.5)	1
8,10	52.8 (47.2,58.4)	1.14***(1.02,1.27)	1.9 (1.6,2.1)	1.38^***^(1.23,1.55)
4,7	64.2 (58.6,69.5)	1.39**^*^(1.23,1.56)	2.5 (2.2,2.8)	1.82^***^(1.59,2.08)
< 4 years	63.9 (56.7,70.6)	1.38^***^(1.21,1.58)	2.7 (2.2,3.2)	1.98^***^(1.64,2.37)
Health services				
Time since last dentist visit				
< 12 months	48.7 (44.5,52.8)	1	1.6 (1.4,1.8)	1
1–2 years	54.5 (49.3,59.7)	1.12^*^(1.02,1.23)	1.8 (1.6,2.0)	1.11 (0.98,1.26)
> 2 years	64.7 (60.0,69.1)	1.33^***^(1.21,1.46)	2.4 (2.2,2.7)	1.50^***^(1.31,1.73)
Has not visited	31.8 (18.0,49.8)	0.65 (0.39,1.09)	0.9 (0.3,1.6)	0.60 (0.31,1.15)
Dental conditions				
DMFT				
0–10	48.7 (42.4,55.1)	1	1.6 (1.3,1.9)	1
11–20	51.6 (47.8,55.4)	1.06 (0.93,1.20)	1.7 (1.5,1.9)	1.07 (0.89,1.30)
> 21	61.0 (55.7,66.1)	1.25^***^(1.12,1.40)	2.4 (2.1,2.7)	1.55^***^(1.33,1.82)
Untreated caries				
0	41.2 (37.2,45.2)	1	1.2 (1.0,1.3)	1
1–3	58.7 (53.5,63.9)	1.43^***^(1.29,1.59)	2.0 (1.7,2.2)	1.68^***^(1.45,1.96)
4–6	63.4 (56.4,69.8)	1.54^***^(1.36,1.74)	2.4 (2.1,2.7)	2.05^***^(1.77,2.38)
> 7	70.8 (60.6,79.3)	1.72^***^(1.47,2.02)	3.4 (2.7,4.1)	2.93^***^(2.31,3.71)
Bleeding on probing				
No	46.8 (42.8,50.9)		1.5 (1.3,1.6)	1
Yes	61.5 (55.8,66.9)	1.31^***^(1.17,1.48)	2.3 (1.9,2.6)	1.54^***^(1.30,1.83)
Dental calculus				
No	46.1 (41.3,50.9)	1	1.4 (1.2,1.6)	1
Yes	58.7 (53.9,63.4)	1.27^**^(1.12,1.44)	2.1 (1.9,2.4)	1.48^***^(1.25,1.74)
Presence of periodontal pockets				
No	48.2 (44.7,51.7)		1.5 (1.4,1.7)	1
Shallow pockets	66.2 (60.3,71.6)	1.37^***^(1.25,1.50)	2.6 (2.2,2.9)	1.66^***^(1.44,1.90)
Deep pockets	73.7 (64.3,81.3)	1.53^***^(1.34,1.74)	2.6 (2.1,3.2)	1.7^***^(1.4,2.0)
Symptoms status				
Dental pain				
No	41.2 (36.6,46.0)	1	1.1 (1.0,1.3)	1
Yes	78.8 (73.6,83.4)	1.91^***^(1.70,2.16)	3.3 (2.9,3.6)	2.93^***^(2.58,3.33)
Capital Social				
Probability of cooperation				
Much or relatively likely	51.0 (47.1,54.9)	1	1.7 (1.6,1.9)	1
Neither likely nor unlikely	59.9 (52.8,66.6)	1.17* (1.04,1.32)	1.9 (1.7,2.1)	1.11 (0.98,1.26)
Much or relatively unlikely	56.3 (50.6,61.8)	1.10 (0.99,1.23)	2.1 (1.8,2.4)	1.21^*^(1.03,1.43)
Feeling of safe				
Much or relatively safe	47.8 (44.3,51.4)	1	1.5 (1.4,1.7)	1
Neither safe nor unsafe	52.8 (44.3,61.1)	1.10 (0.92,1.32)	1.9 (1.5,2.2)	1.22 (0.97,1.53)
Much or relatively unsafe	62.9 (57.6,68.0)	1.32*** (1.20,1.44)	2.3 (2.0,2.7)	1.52^***^(1.30,1.79)
Self-perception of happiness				
Much or relatively happy	51.1 (47.4,54.8)	1	1.7 (1.5,1.8)	1
Neither help nor unhappy	62.1 (53.9,69.5)	1.21** (1.06,1.39)	2.7 (2.3,3.0)	1.59^***^(1.37,1.86)
Much or relatively unhappy	71.9 (58.2,82.4)	1.41*** (1.19,1.65)	3.2 (2.7,3.8)	1.94^***^(1.63,2.31)
Oral health perception				
Self-perception of need of dental treatment				
No	24.2 (20.4,28.6)	1	0.5 (0.4,0.7)	1
Yes	59.8 (56.3,63.2)	2.47^***^(2.11,2.88)	2.1 (1.9,2.3)	3.99^***^(3.20,4.97)
Self-perception of need of dental prosthesis				
No	48.1 (44.5,51.8)	1	1.5 (1.4,1.7)	1
Yes	74.5 (69.7,78.9)	1.55^***^(1.42,1.68)	3.2 (2.9,3.6)	2.13^***^(1.87,2.44)

^a^Proportion (95% confidence intervals) of individuals who answered “yes” to at least one OIDP question. Estimates considered weighting and complex sampling design. ^b^Unadjusted Prevalence ratio and 95% confidence intervals. ^c^Mean of OIDP extent (95% confidence intervals). ^d^Count ratio and 95% confidence intervals. Estimates considered weighting and complex sampling design. ^*^ *p* < 0.05, ^**^ *p* < 0.01, ^***^ *p* < 0.001

Table 5 shows the results of negative binomial regression correlating dentition status with OHRQoL outcomes (OIDP prevalence & extent). For the assessment of SDA, we find the highest adjusted prevalence of OIDP among individuals with < 5 OUs using dental prosthesis, compared to the reference group of those with > 5 OUs/no prosthesis (adjusted PR = 1.26, 95% CI: 1.12,1.43). Concerning OIDP extent, the highest count ratio was found for individuals with < 3 OUs/no prosthesis (CR = 1.77, 95% CI: 1.21,2.59).

**Table 5:** Adjusted association of dentition status with OIDP prevalence and extent in adults. State of São Paulo, Brazil, 2015

	OIDP prevalence		OIDP Extent	
	Unadjusted Prevalence Ratio^a^	Adjusted Prevalence Ratio^a^	Unadjusted Count Ratio	Adjusted Count Ratio
Shortened Dental Arch				
> 5 OUs, no dental prosthesis	1	1	1	1
> 5 OUs, with dental prosthesis	0.96 (0.72,1.28)	0.91 (0.68,1.22)	1.12 (0.79,1.60)	1.17 (0.82,1.67)
3,5 OUs, no dental prosthesis (SDA)	1.20^***^(1.05,1.37)	1.02 (0.91,1.13)	1.58^*^ (1.33,1.87)	1.26^**^(1.09,1.46)
< 3 OUs, no dental prosthesis	1.55^***^ (1.36,1.76)	1.16^*^(1.01,1.33)	2.17^***^(1.67,2.82)	1.77^**^(1.21,2.59)
≤ 5 OUs, with dental prosthesis	1.56^***^ (1.37,1.78)	1.26^***^(1.12,1.43)	2.03^***^(1.47,2.80)	1.64^***^(1.24,2.18)
Not intact anterior region	1.32^***^ (1.22,1.43)	1.09^*^(1.00,1.18)	1.96^***^(1.72,2.23)	1.53^***^(1.34,1.75)
Hierarchical Dental Functional Classification				
Functional dentition, no dental prosthesis	1	1	1	1
Functional dentition, with dental prosthesis	0.89 (0.61,1.30)	0.92 (0.68,1.24)	1.06 (0.69,1.63)	1.26 (0.78,2.03)
No functional dentition, no dental prosthesis	1.38^***^ (1.25,1.51)	1.11^*^(1.01,1.22)	1.72^***^(1.48,1.98)	1.29^***^(1.13,1.48)
No functional dentition, with dental prosthesis	1.34^***^ (1.22,1.48)	1.19^***^(1.10,1.29)	2.04^***^(1.73,2.41)	1.54^***^(1.35,1.74)
WHO criteria for functional dentition				
> 21 teeth, no dental prosthesis	1	1	1	1
> 21 teeth, with dental prosthesis	1.27^***^ (1.13,1.44)	1.13^*^(1.01,1.27)	1.61^***^(1.36,1.90)	1.41^***^(1.22,1.63)
< 21 teeth, no dental prosthesis	1.37^***^ (1.20,1.57)	1.07 (0.95,1.21)	1.99^***^(1.66,2.38)	1.62^**^(1.19,2.20)
< 21 teeth, with dental prosthesis	1.13 (0.95,1.34)	0.95 (0.82,1.09)	1.61^**^(1.21,2.13)	1.38^***^(1.16,1.63)

^a^Prevalence ratio and count ratio (95% confidence intervals). Estimates considered weighting and complex sampling design. ^*^p < 0.05, ^**^p < 0.01, ^***^p < 0.001. Associations were adjusted for years of study and income. Additionally, the final model was adjusted for covariates (sex, skin color, time since last dentist visit, prevalence of untreated caries, safe feeling, self-perception of need of dental treatment, dental prosthesis and dental pain) significantly associated with the outcome (*p* < 0.05)

Turning to hierarchical dental functional classification, we find the highest PR of OIDP problems as well as highest OIDP extent among individuals with no functional dentition plus use of prosthesis (adjusted PR = 1,22. Adjusted CR = 1.54, respectively). Interestingly, for people lacking a functional dentition, the use or nonuse of dental prosthesis did not make a significant difference to OHRQoL, as measured by OIDP prevalence or extent (Table 5).

Lastly, when we turn to WHO criteria, the highest OIDP extent was found for those with < 21 teeth/not using prosthesis (adjusted CR = 1.62). Having fewer than 21 teeth – regardless of the use of dental prosthesis – did not turn out to be correlated with the prevalence of oral impacts (Table 5).

Concerning the type of dental prosthesis, the use of removable partial dental prosthesis was more frequent among those with higher tooth loss (Table 6).

**Table 6:** Proportion of individuals according to dentition status and different dental prosthesis used. São Paulo, Brazil, 2015

Dentition status	Use of dental prosthesis					
	No use		Use of upper or lower FDP^a^		Use of upper or lower RDP^a^	
	n	% (CI)^b^	n	% (CI)^b^	n	% (CI)^b^
Shortened Dental Arch						
> 5 OUs, no dental prosthesis	3471	100.0	0	0	0	0
> 5 OUs, with dental prosthesis	0	0	91	52.9 (39.3, 66.1)	46	47.2 (33.9, 60.8
3–5 OUs, no dental prosthesis (SDA)	487	100.0	0	0	0	0
< 3 OUs, no dental prosthesis	197	100.0	0	0	0	0
≤ 5 OUs, with dental prosthesis	0	0	29	28.7 (17.2, 43.7)	88	71.4 (56.3,82.8)
No intact anterior region	537	37.7(33.6,42.0)	77	4.1 (2.7, 6.0)	730	58.2 (53.4,63.0)
Hierarchical Dental Functional Classification						
Functional dentition, no dental prosthesis	2805	100.0	0	0	0	0
Functional dentition, with dental prosthesis	0	0	73	59.2 (41.4,74.9)	22	40.8 (25.1, 58.6)
No functional dentition, no dental prosthesis	1887	79.8(77.7,81.7)	0	0	0	0
No functional dentition, with dental prosthesis	0	0	124	9.8 (6.9, 13.8)	842	90.2 (86.2, 93.1)
Who criteria for functional dentition						
> 21 teeth, no dental prosthesis	4452	100.0	0	0	0	0
> 21 teeth, with dental prosthesis	0	0	172	23.9 (18.3,30.6)	359	76.0 (69.4, 81.7)
< 21 teeth, no dental prosthesis	240	100.0	0	0	0	0
< 21 teeth, with dental prosthesis	0	0	25	4.2 (2.3,7.6)	505	95.8 (92.4,97.7)

^a^*FDP* Fixed Dental Prosthesis, *RDP* Removable Partial Dental Prosthesis. ^b^ 95% CI (95% confidence. Estimates considered weighting and complex sampling design intervals)

## Discussion

Adults with more missing teeth and a poorer dentition status had a higher impact on OHRQoL, as assessed by OIDP prevalence and extent, regardless of the use of dental prosthesis, and irrespective of the definition of dentition status. This finding is the most relevant result of the current study. Previous studies have already reported the association between tooth loss and OHRQoL [6, 31–33]. Reduced masticatory efficiency and chewing ability, changes in dietary intake, aesthetic and psychosocial problems caused by missing teeth can explain this association [13]. Systematic reviews concluded that the retention of teeth is associated with better OHRQoL [6, 33], and that this association occurred regardless of the OHRQoL assessment tool and the background context of the population [6]. Tooth loss was a strong predictor of changes in Oral Health Impact Profile scores, another OHRQoL index, in a two-year longitudinal study [32].

The current study also observed that OHRQoL depends on the number of occluding pairs and the location of remaining teeth, which is consistent with previous literature reports [6, 31, 33, 34]. Previous studies also support the current observation of a shortened, functional dentition to be compatible with favorable outcomes in patient-centered measures of oral health [7–10, 19]. Not all missing teeth have the same adverse effect on physical and psychosocial well-being [33]. A meta-analysis showed a direct correlation between the number of remaining teeth and OHRQOL impacts, with a marked deterioration once the number of teeth drops below 20. The number of natural occluding pairs has also been correlated with OIDP impacts [6]. This study took account of the numbers of natural posterior occluding pairs and retention of the anterior region. We also considered solely counting the number of remaining teeth because the WHO adopted this criterion in the definition of global goals for oral health, and several studies in Brazil used it [8, 18, 35].

Individuals with SDA (3–5 OUs, no dental prosthesis) did not report higher OIDP prevalence than those with a higher number of OUs (> 5 OUs, no dental prosthesis) as previously observed in Brazil and Australia [10, 17], reinforcing the conclusion that shortened dentition is compatible with unimpaired OHRQoL [36, 37]. Both epidemiologic studies [10, 17] concluded that, despite having several missing teeth, many adults are still able to keep a functional daily living without dental prosthesis, which challenges the prevailing clinical approach of replacing any missing tooth with prostheses. Similar to this study, higher OIDP extent in SDA group was also observed among Australians individuals (> 15 years old) [17]. The OIDP extent score is derived from number of items with non-zero score on nine different domains of oral health and, thus, may be more sensitive than the prevalence measure (at least one impact) when investigating the effect of the quantity and location of remaining teeth on quality of life [6].

This study showed that OIDP prevalence and extent did not differ between those who used or did not use dental prosthesis if the anterior region is intact and a higher number of occluding posterior units are preserved. By contrast, adults with more missing teeth (no functional dentition, < 5 OUs, or < 21 teeth) had higher OIDP prevalence and extent, even if they used dental prosthesis. These results suggest that dental prosthesis can fail to improve OHRQoL among adults with a severely affected dentition status. Along with this line, a previous Chinese cross-sectional study concluded that from the OHRQoL perspective, natural teeth are preferred over artificial teeth. The authors compared the effect of prosthetic tooth replacement and showed that individuals with fixed and removable dental prosthesis had significantly higher odds for impaired OHRQoL than their counterparts with similar dentition, though with more natural teeth [7].

The literature has also highlighted that the effect of removable dentures on how well patients perform may not be predictable and can even give rise to additional problems [38, 39]. As the use of removable partial dental prosthesis was the most frequent in the sample examined here, the type of prosthesis may have influenced the findings. However, the quality of the prosthesis was not assessed in this study; we cannot rule out that ill-fitting removable dentures may have caused pain, discomfort, and negative oral impacts. A recent systematic review assessed different dental prosthetic interventions and changes in OHRQoL [40], including clinical trials and cohort studies, and showed that FDP had short, and long-term positive effects on OHRQoL, whereas RPD positively affected OHRQoL in the short term, though not after nine months. According to the authors, the lack of effect of RPD after nine months could be due to issues concerning maintenance, distortions in the fit over time, adverse effects on periodontal health, or changes in outcome expectation [40]. Furthermore, previous studies have already reported that RPD has a higher likelihood of success when they replace anterior teeth [41].

In our study, individuals with > 21 teeth who used dental prosthesis had a higher OIDP prevalence and extent than those with the same number of remaining teeth, though without a dental prosthesis. The fact that nearly 80% of the former group used removable partial dental prostheses may have influenced this observation. Rehabilitation with RDP does not guarantee a positive impact on OHRQOL [40]. Similarly, a previous study in Finland reported that among adults with 20 or more teeth, those wearing RDP were more likely to report oral impacts than those who did not [42].

The number and position of missing teeth can influence how patients perceive the need for dental prosthesis [43]. In this study, the effect of dental prosthesis on OHRQoL varied according to the definition of dentition status. For the assessment of SDA and the hierarchical dental functional classification system, the use of dental prosthesis among those with a higher number of natural teeth was not significantly associated with OHRQoL. For the WHO criteria, the use of dental prosthesis had a negative impact on OHRQoL when the comparison is restricted to those with > 21 teeth. Merely counting the number of teeth seems to be an overly simplistic definition for the description of oral functionality [25]. A previous Brazilian study showed that 54.7% of the adults with > 21 teeth met all criteria to functional dentition according to the hierarchical dental functional classification system and the concordance between the two criteria was low (kappa = 0.32) [25].

Having assessed a large and representative sample of adults in the most populous Brazilian state, and having gathered data following methods standardized by the WHO [33] are strengths of this study. The outcome variable, OHRQoL, is a patient-centered measure that should be included in the decision-making process regarding tooth extraction or retention, and choice of any treatment modality [33]. Our statistical assessment also took into account sample weights and the complex sampling design. Examiners were not aware of our hypotheses. Hence, our findings are unlikely to have been affected by misclassification, interview or selection bias. A relevant study limitation is its cross-sectional design, which does not allow to order in time the study outcome and its main covariates. The exclusion of individuals using bimaxillary prosthesis may have underestimated the prevalence of adults with the worst dentition status (with dental prosthesis). Based on our cross-sectional analyses, we cannot conclude if the use of dental prosthesis is or is not related with improved OHRQoL. Our results suggest that the association between dental prosthesis and quality of life depends on the remaining dentition status as well as other clinical variables, which were not assessed in this study, although they can affect oral impacts, such as the quality of fit and time of experience with a current dental prosthesis.

## Conclusions

The findings of this study reinforce previous evidence showing that the retention of natural teeth is associated with less oral impacts and that dental prosthesis is not always associated to a higher quality of life. The effect of dental prostheses on OHRQoL varies according to the number and location of remaining teeth. Therefore, the decisions about restorative treatments and prosthetic replacement should include patient-centered assessments in addition to the professional judgment.

## Abbreviations

FDI: FDI World Dental Federation
FDP: Fixed Dental Prosthesis
OHRQoL: Oral health-related quality of life
OIDP: Oral Impacts on Daily Performance
OU: Occlusal Unit
OUs: Occlusal Units
POP: Posterior Occluding Pairs
RDP: Removable Dental Prosthesis
SDA: Shortened Dental Arch
WHO: World Health Organization
